# ChildWeCare: An Innovative System for the Surveillance and Care of Early Childhood Development Disorders in Thailand

**DOI:** 10.3390/children12040522

**Published:** 2025-04-18

**Authors:** Duangkamol Tangviriyapaiboon, Chayut Owatsakul, Patrinee Traisathit, Salinee Thumronglaohapun, Pimwarat Srikummoon

**Affiliations:** 1 Rajanagarindra Institute of Child Development, Chiang Mai 50180, Thailand; duangkamol@dmh.mail.go.th; 2 Science and Mathematics, Chiang Mai University Demonstration School, Chiang Mai 50200, Thailand; 3 Department of Statistics, Faculty of Science, Chiang Mai University, Chiang Mai 50200, Thailand

**Keywords:** innovation, visualization, telehealth, child developmental delay, early intervention, Thai children

## Abstract

Background: A structure survey conducted by the Department of Health on early childhood development in Thailand indicates that 27.20–32.50% of preschool children have developmental delays. These children require appropriate care and constant stimulation to help them develop normally. Methods: The ChildWeCare innovation system for monitoring children and providing appropriate care for those with developmental delays from birth to 5 years of age has been developed. This is accompanied by qualified personnel providing assistance to parents for their child’s development. Enrollment of participants and provision of services via the system were implemented. Appropriate intervention from the ChildWeCare system will be provided for each specific child, and each parent will be assigned homework for training their children. Results: The database of the ChildWeCare system was developed using MySQL, which can store information about the child, parent, homework, and log usage system, as well as data on each parent’s homework assignments. Our pilot testing shows that parents were satisfied with the ChildWeCare system. Conclusions: The ChildWeCare system could provide guidance on suitable stimulation techniques and strategies for each child’s specific needs. These preliminary results could indicate the advantages of further plans for the system’s implementation in other settings in Health Region 1 or nationwide.

## 1. Introduction

Early childhood (from birth to five years old) is an extremely important period in a child’s physical, emotional, mental, social interactional, and intellectual development [1,2]. It has recently been reported that the number of children with developmental disorders, such as attention deficit hyperactivity disorder (5–11%), learning disorders (3.5%), and autism spectrum disorders (2%), is dramatically increasing worldwide [3,4]. Moreover, there are higher prevalences of autism and intellectual disabilities in children in Thailand than in other Southeast Asian countries [5,6], which could be due to the higher prevalence of low birth weight and/or asphyxia during birth [7]. Hence, early childhood screening and surveillance can play a crucial role in identifying developmental problems and providing appropriate developmental stimulation from an early age, thereby enhancing the IQ and EQ scores of affected children.

According to a survey by the Department of Health conducted in Thailand between 1999 and 2017, 27.20–32.50% of early childhood development interventions led to inappropriate care [8,9]. Children with developmental delays need appropriate care and constant developmental stimulation to engender self-reliance and lessen the burden on caregivers and family members. In accordance with the Development Surveillance and Promotion Manual (DSPM) in Thailand, the development of children is assessed at 9, 18, 30, 42, and 60 months of age to track their progress, with a focus on childcare, monitoring, screening, and encouraging their development. Children diagnosed with developmental delays during the first diagnosis are referred immediately for appropriate treatment, while those at risk of developmental delays are provided with further screening. In cases of developmental delays in at least one domain, children receive developmental stimulation to help them as they age [10].

The Ministry of Public Health reported that approximately 1.8 million Thai children under five years of age had their first DSPM assessment in 2018. Of these, 7970 children had developmental delays, both in the first DSPM assessment and in the second assessment using the Thai Early Developmental Assessment for Intervention (TEDA4I) instrument. However, only 3676 children received appropriate developmental stimulation, while 4294 children were lost to follow-up. In addition, in 2019, 2020, and 2021, the number of lost-to-follow-up cases nationwide was reported as at least 2539, 3415, and 3758 children, respectively [11]. Indeed, there could be even more children with developmental delays who have not been assessed and are not receiving proper care and developmental stimulation for development.

High rates of loss to follow-up after a developmental delay diagnosis have been reported for eight provinces in Health Region 1 in northern Thailand (Chiang Mai, Lamphun, Lampang, Phrae, Nan, Phayao, Chiang Rai, and Mae Hong Son): 1223, 811, and 522 in 2020, 2021, and 2022, respectively (data as of October 2022 [11]). Of these, 23.65–27.25% had not been tracked using the current monitoring system and thus had not received appropriate developmental stimulation. In 2021 and 2022, the parents or caregivers of children who did not regularly attend appointments reported that the main reason was their availability (90%), followed by travel costs (59%) and COVID-19 (59%). Most of them (87%) wanted their child to receive developmental stimulation. They also suggested that online monitoring and appointment reminders would be advantageous and that developing a handbook for caregivers and providing assistance with monitoring and developmental stimulation (e.g., text reminders, phone or video calls, and home visits) would be helpful [12,13,14,15,16].

Over the past decade, internet coverage and the number of internet users in Thailand has dramatically increased. The National Broadcasting and Telecommunication Commission of Thailand reported that approximately 52.16 million people had access to the internet in 2021 [17]. In addition to personal and business purposes, internet usage by government agencies for tasks such as online booking, documentation, and telehealth services has also increased. Since the outbreak of COVID-19, telemedicine has become an alternative and popular healthcare service in Thailand. In 2023, the Line application was being actively used by approximately 77% of mobile internet users in Thailand [18]; it is the most popular free chat application in Thailand and has become an important online platform for healthcare providers to provide telehealth services. It enables users to exchange text messages and share photos or videos, thereby enabling convenient and efficient communication between clients and healthcare providers. The outcomes from a previous study of a Line application for telehealth consultation for contraceptive implants during the COVID-19 pandemic in Thailand revealed high client satisfaction with self-managing any adverse effects from the procedure [18].

The main reason for the loss to follow-up and withdrawal from the developmental stimulation program of children with developmental delays has been the availability of their parents. Thus, our aim is to provide a service that could help parents to continue developmentally stimulating their children even when they cannot attend follow-up visits. We hypothesized that providing developmental stimulation resources and communicating with parents via a Line application might be advantageous for the treatment of children with developmental delays. Therefore, our aim was to develop the ChildWeCare system to monitor and provide care for children with developmental delays via Line. After screening using DSPM, parents of children with developmental delays receive follow-up appointments for child developmental stimulation. This innovative system for monitoring patients could help to minimize the number of missing or lost-to-follow-up cases. Furthermore, childcare and development are also provided through this innovative system. Parents can gain knowledge and suggestions for taking care of their children and be trained in how to stimulate their children’s development appropriately. In addition, parents can contact the healthcare providers through the system during office hours to ask questions or seek advice related to child development.

## 2. Materials and Methods

### 2.1. Study Design and Conceptual Framework

**Figure 1:**
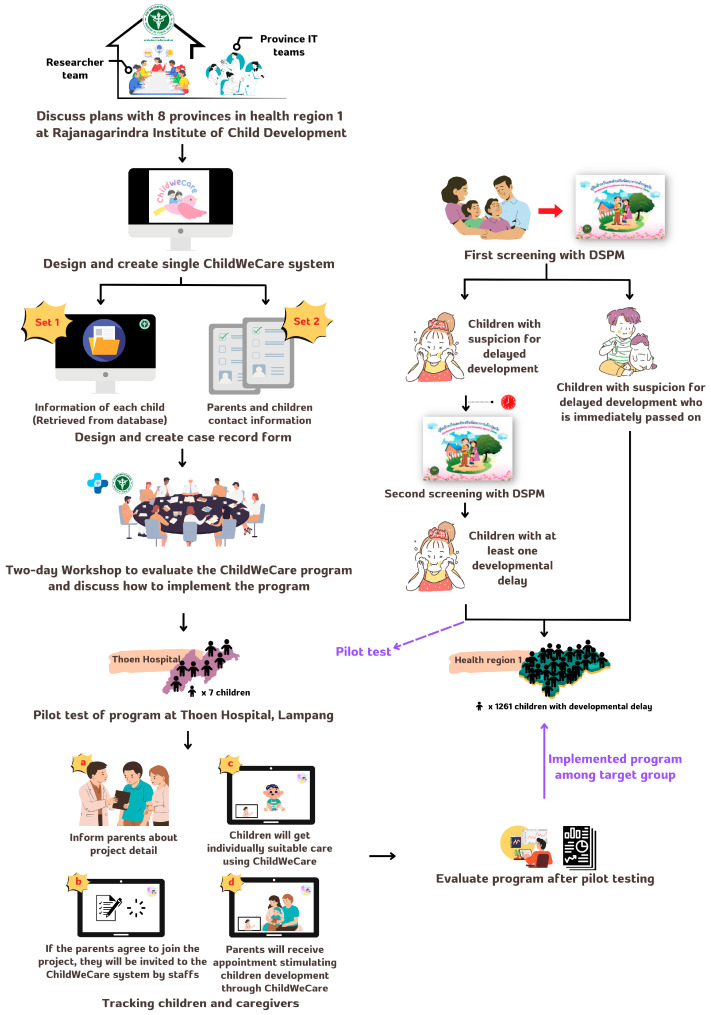
The conceptual framework for the ChildWeCare system.

This was a developmental study to assess the ChildWeCare system for tracking and providing care and counseling for caregivers of children with developmental delays identified by screening using DSPM or TEDA4I. As presented in Figure 1, the ChildWeCare developmental framework was separated into two phases as follows.

(1) Program development

In this phase, the research and IT teams first discussed the development plans with healthcare staff from all eight provinces in Health Region 1 at the Rajanagarindra Institute of Child Development. Subsequently, the program development team designed and created a single ChildWeCare system to link the information of each child with the Health Data Center (HDC) Service database, which includes data on all public health activities from all the hospitals and healthcare service units throughout the country (https://hdcservice.moph.go.th, accessed on 20 December 2023). Parents’ and children’s contact information and case record forms were also designed and prepared. After finishing the design of the program, the staff attended a two-day workshop to evaluate the quality and content validity of the program prior to pilot-testing the program.

(2) Program pilot testing

This study was conducted at Thoen Hospital, Lampang. The eligibility criteria for the participants were that the children were aged under five years old and had been diagnosed with a developmental delay in at least one domain according to the DSPM or TEDA4I. The staff informed the parents about the project details, and then the parents received an appointment through the ChildWeCare program after registration. The parents and children received suitable care via ChildWeCare, and their attendance was tracked. The efficiency of the program and user satisfaction were evaluated after finishing the pilot test.

### 2.2. Settings and Target Group

The ChildWeCare system was developed and preliminarily tested on volunteer caregivers of children with developmental delays in the first assessment or at least one domain of developmental delay in the second assessment. System development and pilot testing were conducted in 2023–2024 in eight provinces in northern Thailand (Health Region 1), including Chiang Mai, Lamphun, Lampang, Phrae, Nan, Phayao, Chiang Rai, and Mae Hong Son. The target group for the ChildWeCare program was 1261 children with developmental delays according to the DSPM assessment in this area. After the pilot test, we plan to enroll these children and their parents for further evaluation of the program.

### 2.3. Child Developmental Delay Assessment

Developmental milestones are a set of goals or markers that a child is expected to achieve during maturation [19]. These are categorized into five domains: gross motor (GM), fine motor and intelligence (FM), receptive language (RL), expressive language (EL), and personal and social (PS) skills [20,21].

### 2.4. Care and Promotion of Early Intervention Childhood Development in Thailand

In 2013, the Rajanagarindra Child Development Institute, Department of Mental Health, Ministry of Public Health, Thailand, won first place in the United Nations Public Service Awards 2013 in the category “Improving the Delivery of Public Services” as part of the “Child First Work Together” project. The aim is to constantly support children in their growth and development by developing a process for screening, evaluating, and promoting child development. Empirical evidence from the publication of international academic articles suggests that this child developmental system provides the largest scope of care in the world [22].

The Department of Mental Health, Ministry of Public Health, has developed tools for screening, diagnosing, and providing services to help children with developmental disorders throughout the country, and the number of children provided with the service is rapidly increasing [23]. Moreover, significant improvements in monitoring children with suspected developmental delays have been achieved over the past decade [21].

### 2.5. Child Development Screening Tools Used in Thailand

Currently, large provincial or district hospitals in Thailand can screen child development with basic tools, such as the Developmental Assessment for Intervention Manual (DAIM), DSPM, or TEDA4I. DAIM comprises a manual for early monitoring and promoting the development of children, especially those who have suffered birth asphyxia and/or low birth weight who are at risk of intellectual disability, developmental delay, and/or learning problems. The tool is used along with physical examination until the child reaches 2 years old, after which DSPM is used for further assessment. The DAIM tool covers the five development domains mentioned earlier. If a child’s development is found to be impaired for their age, an appointment for re-evaluation will be scheduled within one month. If the diagnosis is the same, they will be referred immediately [24,25,26,27,28].

DSPM is a manual for monitoring and promoting early childhood development (from birth to 5 years of age) in the following developmental skill domains: (i) gross motor (GM), (ii) fine motor (FM), (iii) receptive language (RL), (iv) expressive language (EL), and (v) personal and social (PS) skills (sensitivity = 96.04%; specificity = 64.67%) [21]. It was developed based on the Thai Developmental Screening Inventory (TDSI), Denver II instrument used by the Queen Sirikit National Institute of Child Development. It consists of 116 items in the five domains, including 24 for GM, 24 for FM, 22 for RL, 22 for EL, and 24 for PS. It is divided into 19 age ranges (15 for surveillance and 4 for screening). The process involves observing the child at play, testing with the methods in the manual, and interviewing the caregiver [8].

If a child fails testing in one of these domains, they are referred for another assessment conducted using the TEDA4I (validity = 0.84; reliability = 0.97) [21]. This is an assessment guide to help children with developmental problems developed from TDSI, Denver II by the Queen Sirikit National Institute of Child Development. It consists of 145 items in the five domains: 25 for GM, 29 for FM, 29 for RL, 34 for EL, and 28 for PS. Each item is divided into 18 age ranges. This tool is used if the result of the DSPM assessment shows that the child has developmental delays and did not recover after having been treated for 1 month. The assessment is carried out through observation and assessment of the child according to the manual and interviewing the caregiver [29].

### 2.6. System Development and Workflow

This innovative system was developed to integrate databases, telehealth services, and technology. The ChildWeCare system is linked to the Thai national database, Thai child development, and the proposed monitoring system via the Line app. The development team designed the system platform, system interfaces, and record form for the system using the Vscode program for system development and design. The database was developed using the Structured Query Language (SQL), which is the main programming language for managing data stored in databases. While SQL was initially used only with relational database management systems, its use has been significantly extended with the advent of new types of database systems. Specifically, SQL is a powerful query language for highly distributed and scalable systems that process big data (i.e., datasets with high volume, velocity, and variety). It can be divided into two main categories. First, the Data Definition Language (DDL) is used to manage tables and index structures. The most basic items of DDL are the CREATE, ALTER, RENAME, and DROP statements. Second, the Data Manipulation Language (DML) is a subset of SQL used to add, update, and delete data in tables [30,31].

**Figure 2:**
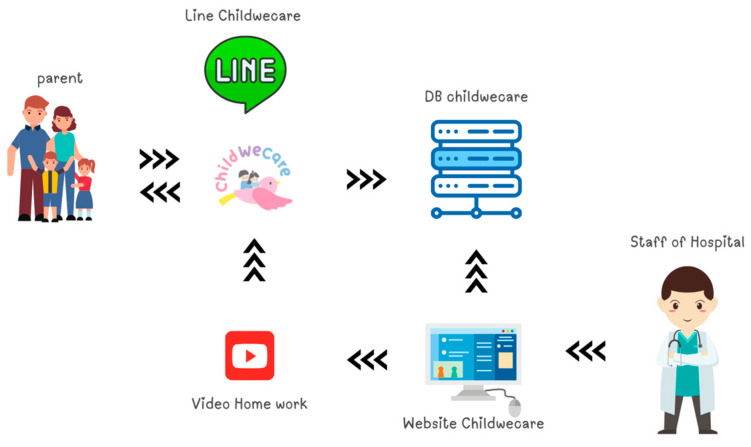
The workflow for the ChildWeCare system.

We used the MySQL program to store participant information and for record management and retrieval. In addition, we applied data visualization to present the information and results of the assessment. Data visualization is a powerful tool for enhancing understanding and communication of complex data that has the ability to represent a vast amount of data immediately and enables viewers to identify emergent properties to enhance understanding of data [32,33]. The workflow for the ChildWeCare system is presented in Figure 2.

### 2.7. The Structure and Components of the ChildWeCare System

#### 2.7.1. The Parent Interface

**Figure 3:**
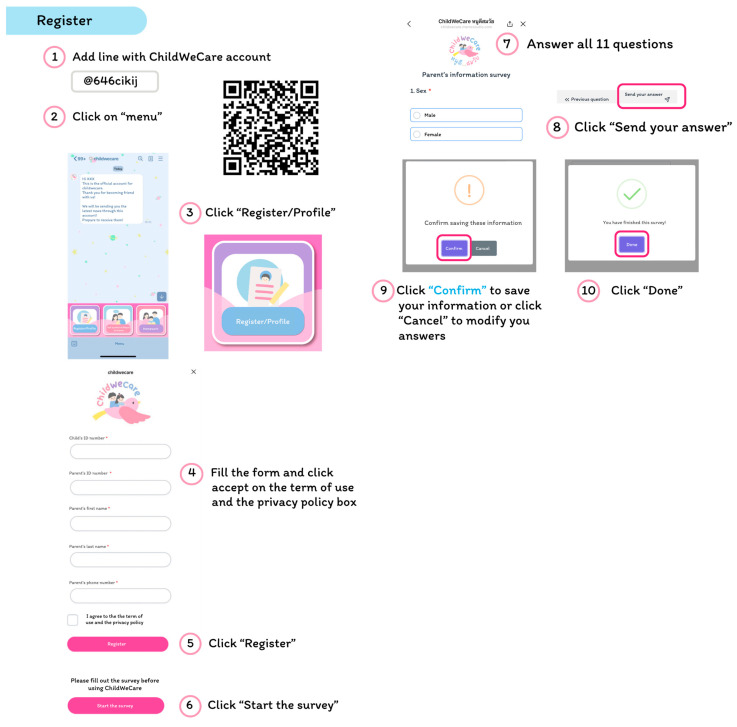
The registration interface.

Parents are asked to add their official Line IDs to register their account for ChildWeCare. They also fill in their personal information, phone number, their child’s information, and a short demographic survey on the registration page (Figure 3).

**Figure 4:**
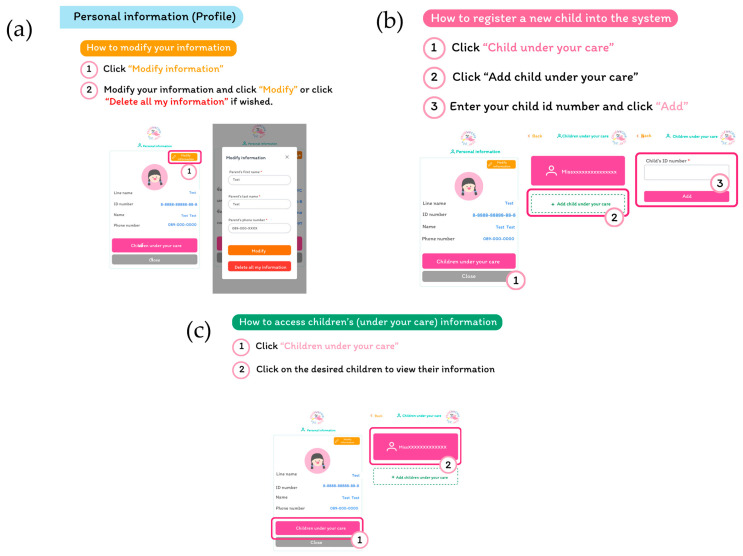
The personal information interface. (**a**) modify information interface (**b**) register a new child interface and (**c**) access their child’s information interface.

After registration, parents can check and modify their information if necessary, and they can also delete their information at any time (Figure 4a). In the personal information interface, parents can add a new child under their care into the system (Figure 4b) or access their child’s information (Figure 4c).

**Figure 5:**
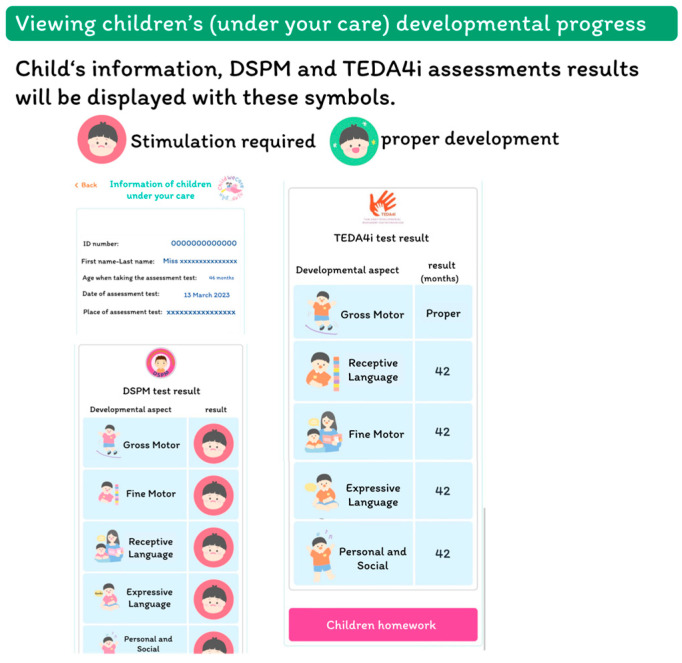
The children’s developmental progress interface.

**Figure 6:**
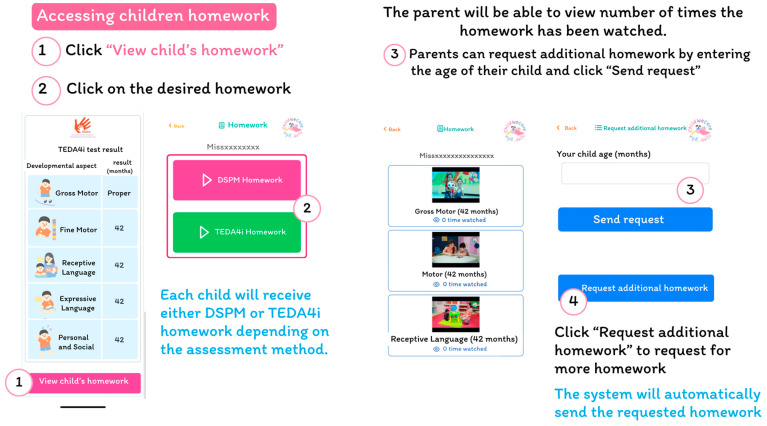
The children’s homework assignment interface.

The interface to access the child’s information is presented in Figure 5, in which the child’s details, DSPM or TEDA4I assessment results, and developmental ages for each domain are shown. The assessment results and the developmental stimulation assignments will be automatically assigned to the caregivers of the children within one or two days of receiving the assessment results. The parent can access the child’s homework assignments depending on the assessment methods and results, view the length of each homework assignment, and request additional ones if desired (Figure 6).

**Figure 7:**
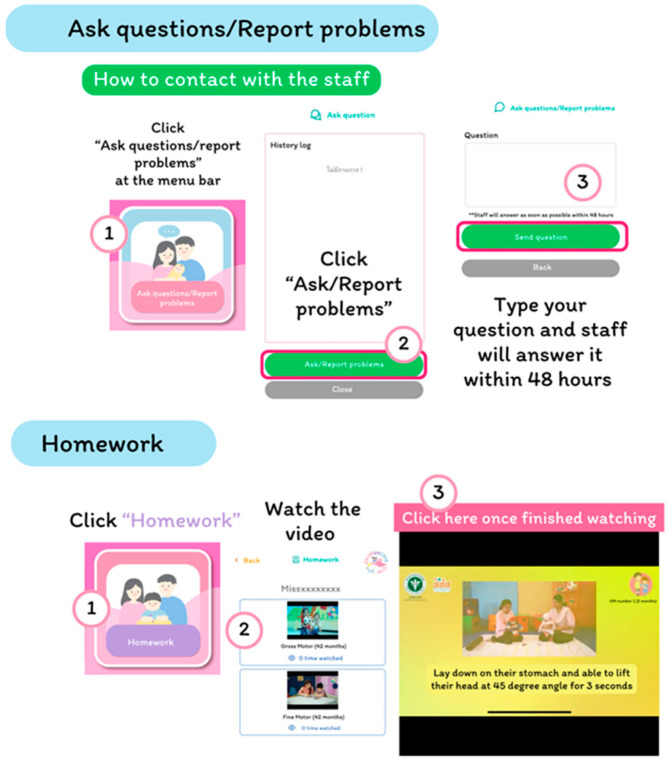
The inquiry interface.

Parents can inquire about or report problems with using the system at any time (Figure 7). Staff should answer their questions within two business days.

#### 2.7.2. The Hospital Staff Interface

**Figure 8:**
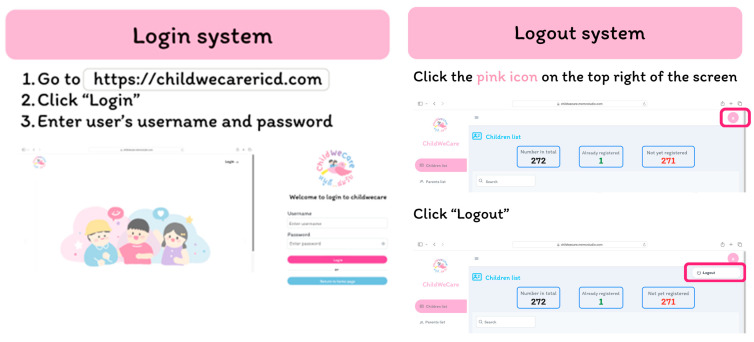
The hospital staff login and logout interface.

Hospital staff can log in and out of the system via https://childwecarericd.com. The login requires a username and password (Figure 8).

**Figure 9:**
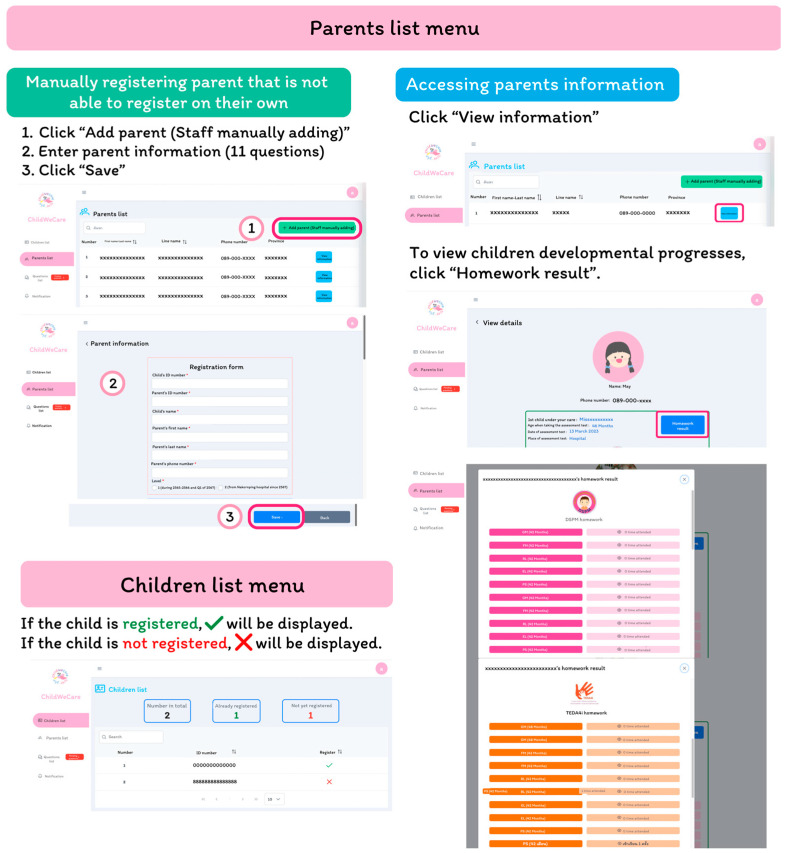
The parents’ and children’s list interface.

The hospital staff can view the registered information of parents and children, including the status of the registration, personal information, developmental progress, and homework assignment results. They can also manually register parents who could not register by themselves (Figure 9).

**Figure 10:**
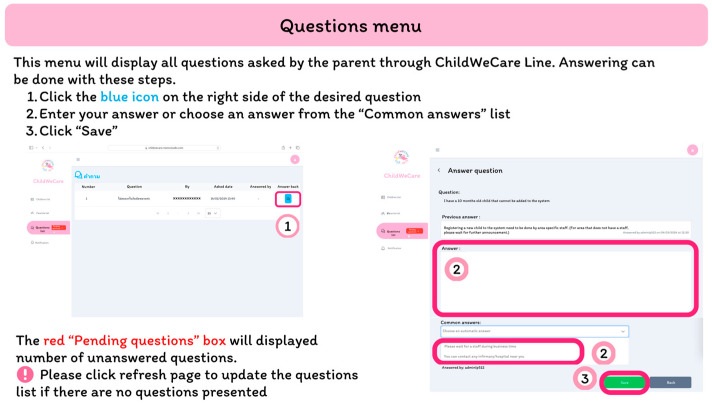
The user support interface.

The user support interface displays a list of questions from the parents. A staff member can answer the question or set as “pending” unanswered questions that need to be answered by other staff members via this interface (Figure 10).

**Figure 11:**
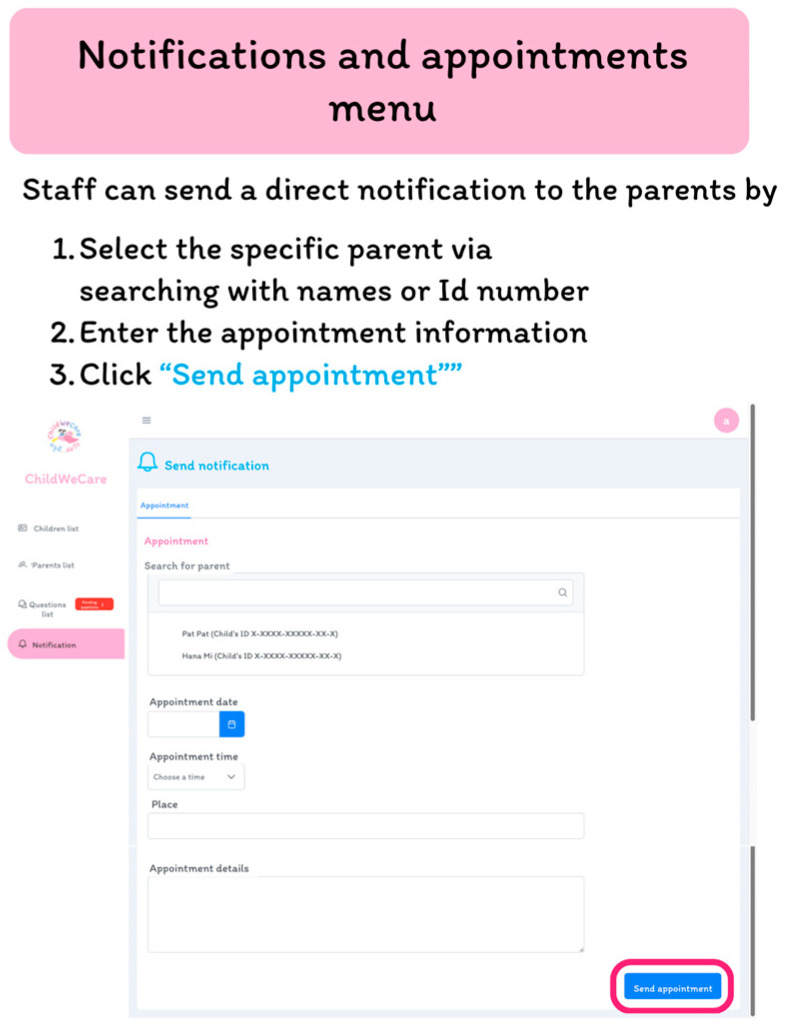
The notifications and appointments interface.

The staff can send direct notifications to the parents by selecting the name or ID number of the latter (Figure 11).

### 2.8. The ChildWeCare Database

**Figure 12:**
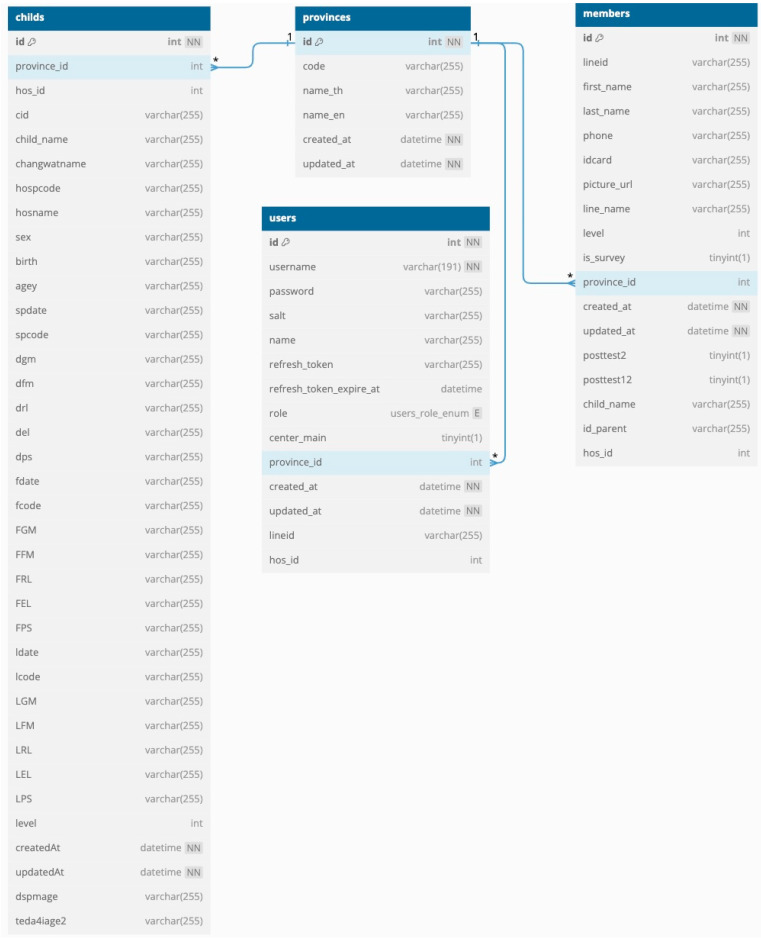
A schema showing the relationships between child, user, member, and province tables. Note: 1 and * refers to its being primary key and foreign key, respectively.

**Figure 13:**
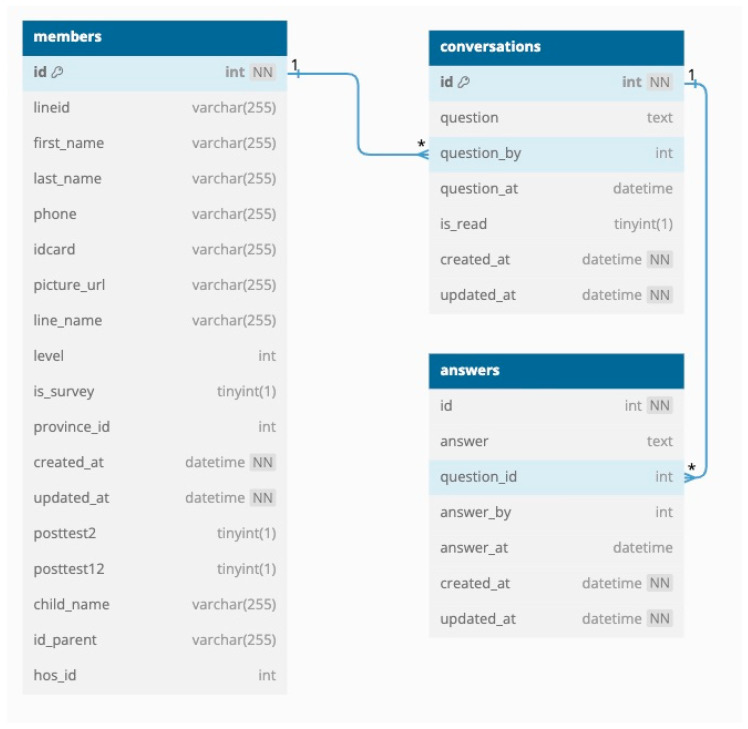
A schema showing the relationships between member, conversation, and answer tables. Note: 1 and * refers to its being primary key and foreign key, respectively.

**Figure 14:**
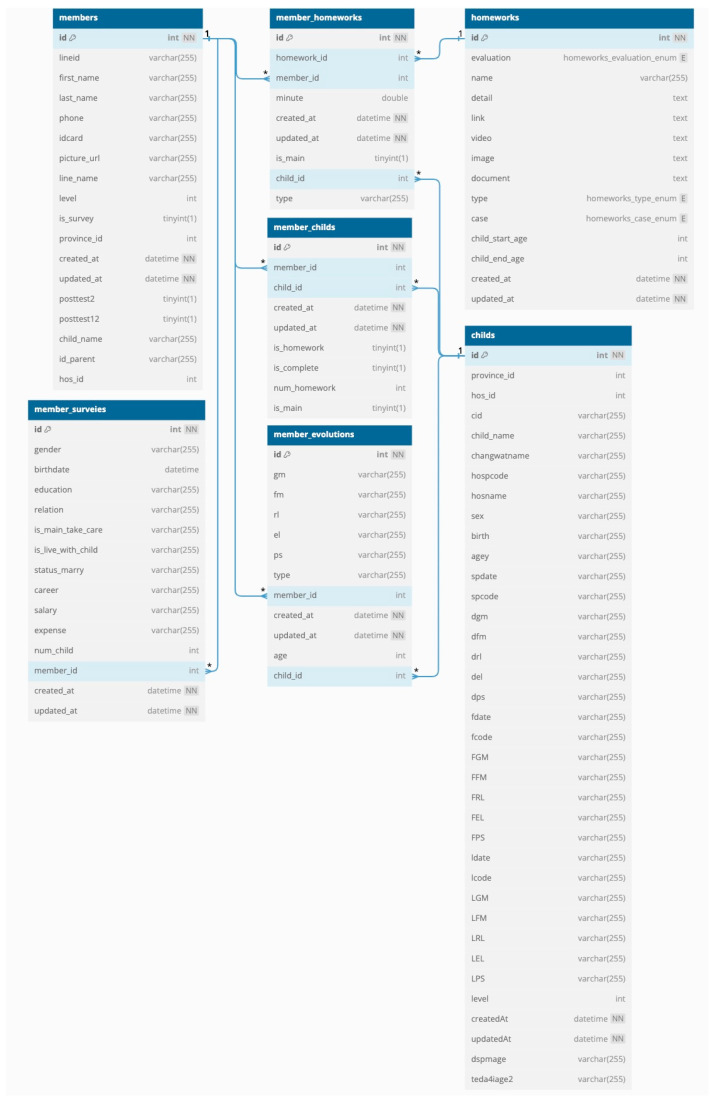
A schema showing the relationships between child, member, homework assignment, member_survey, member_homework, member_child, and member_evolution tables. Note: 1 and * refers to its being primary key and foreign key, respectively.

The database was developed using MySQL, which can store data in various table formats and handle large volumes of data. The database can store information about the child, parent, outstanding and completed homework assignments, and usage. It comprises 11 tables, including children (child information and DSPM and TEDA4I results), members (parent information), users (hospital staff), provinces (province name), conversations (questions from parents), answers (answers from staff to the questions from parents), homework assignments (details such as the name and age of the child, the type of homework, DSPM or TEDA4I assessment information, and video links), member_surveys (survey answers from the parents when registering on ChildWeCare), member_homework (the relationship schema for homework assignments and members), member_child (the relationship schema for children and members), and member_evolution (the relationship schema for evolution and members) (Figure 12, Figure 13 and Figure 14).

### 2.9. Homework Assignments for Parents and Children in the ChildWeCare System

The assignments provided for parents are based on the assessment tool used (DSPM or TEDA4I), the outcome of the assessment, and the child’s age. For example, a boy who is 20 months old had gross muscle development failure at 18 months old but not at 15 months old. This means that the boy is actually 20 months old but has a developmental age of 15 months old. Therefore, his parents will be allocated a homework assignment for the GM domain including a video clip from Table 1 (the homework items for DSPM) or Table 2 (the homework items for TEDA41).

From Table 3, Parents 1, 2, and 3 will receive 6, 9, and 9 homework assignments, respectively.

### 2.10. System Pilot Testing

The pilot test of the innovative ChildWeCare system was conducted during the “Take Doctors to Meet People” event on 3 March 2024 at Thoen Hospital, Lampang. According to the recruitment, seven parents voluntarily participated in this pilot test phase. The enrollment of participants and provision of services via the system were implemented via the following steps:

(1) Information and related details were clarified for the caregivers prior to enrolling in and accessing the system.
(2) Caregivers were instructed on how to use the ChildWeCare system.
(3) Appropriate intervention from the ChildWeCare system was provided for each child and homework assignments were provided to the parents to train their children.
(4) Follow-up appointments were sent to the parents and usage data were recorded.
(5) Contact and inquiry were enabled through the system for parents who needed help or had questions about their child’s development. These were answered by a healthcare official within two business days.

## 3. Results

### 3.1. The Validation of the ChildWeCare System

After finishing the preparation of the system, we conducted a two-day workshop to evaluate its validity and discuss its implementation with relevant staff in the eight provinces in Health Region 1. The content of the developmental stimulation program was approved by all of the staff members. However, there were some suggestions related to the registration and children’s information interfaces, including adding “Unknown” as a choice for household income and the date of developmental assessment and providing clearer graphics. In addition, the staff also suggested that, since problems with internet access can occur, paper-based supporting documents should be prepared.

### 3.2. Demographic Data of Children Enrolled in the ChildWeCare System during the Pilot Test

During the “Take Doctors to Meet People” event at Thoen Hospital, Lampang, many of the parents concerned about their child’s development came for a pediatrician visit. Some of them worked abroad and the grandparents who took care of their children lacked knowledge about how to treat the child’s disability, while some were single parents whose children were in temporary care with neighbors during the daytime. In these cases, taking children for developmental screening and treatment at a hospital is very challenging. Moreover, the number of child development specialists was limited, resulting in fewer opportunities for regular assessment and treatment.

During this event, the ChildWeCare team provided services to seven children who were assessed as needing early childhood development by nurses with expertise in using the TEDA4I tool. In most cases, the disability had already shown but their parents did not know where to seek the service. Some of the children had severe developmental delays and needed to be referred for care and treatment at the Rajanagarindra Child Development Institute, Chiang Mai. The health provider team realized that the regular referral process took a long time after their delayed development had been confirmed. Thus, they introduced the parents to the ChildWeCare system and invited them to enroll for appropriate and immediate intervention. Each parent registered and input their child’s information, and then they received an appropriate assignment to prepare their children while waiting for the referral. Of the seven children who participated in this pilot test, the maximum developmental age was 3 years old. Interestingly, one child had been diagnosed very late (at almost 8 years of age) whereas her developmental age was only 3 years old (Table 4).

### 3.3. Assignment Workflow in the Pilot Test

The characteristics of the caregivers who voluntarily participated in the pilot test are provided in Table 5. Although their educational levels varied, this did not affect the registration for and receipt of developmental stimulation assignments for their children.

### 3.4. Systems Usage and Satisfaction

**Figure 15:**
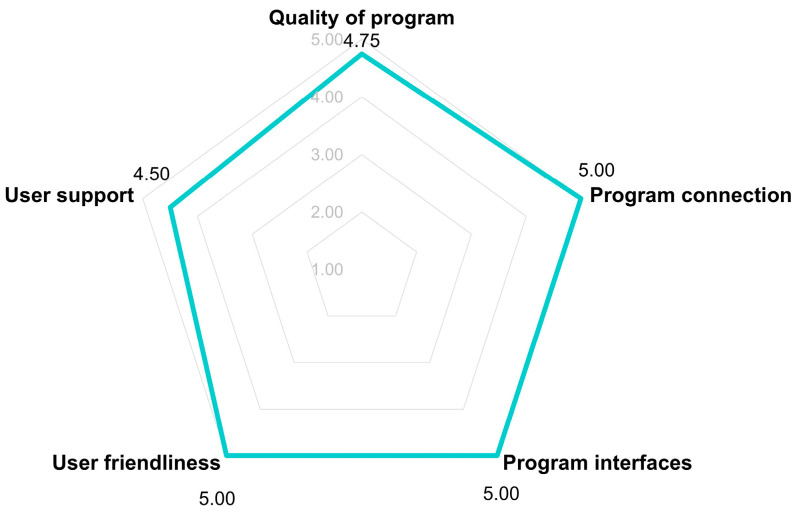
Satisfaction of the parents with the ChildWeCare system.

All of the parents frequently accessed the ChildWeCare system after returning home (average: 3–4 times per week). After participating in the pilot testing of the system for 8 weeks, the parents were contacted and asked about their satisfaction with using the system, including the quality of the program, program interfaces, program features, user-friendliness, and user support. Each domain was evaluated using a Likert scale from 1 (very dissatisfied) to 5 (very satisfied). The results (four of seven parents responded) show that the satisfaction with each item was high on average (Figure 15).

### 3.5. User Problems or Requests

We also asked the parents about any problems in accessing the ChildWeCare system, to which the parents responded that there were none. There was one instance where the parent later contacted the service via ChildWeCare chat after returning home to request additional registration for another child, and four parents requested additional homework. This could reflect that the parents saw the benefits of using the ChildWeCare system. However, further testing with a much larger number of users is necessary.

### 3.6. SWOT Analysis

After the pilot testing of the ChildWeCare system, we discussed its strengths, weaknesses, opportunities, and threats (SWOTs) related to the participants’ demographics and program usage information (Table 6).

## 4. Discussion

The ChildWeCare system can help to address poor service for children with developmental delays by providing parents with knowledge and suggestions, including training methods for effective stimulation development that are suitable for each child and making or rescheduling hospital appointments at the parents’ convenience. Moreover, the child’s general information and developmental screening history, such as gender, weight, height, and screening results, will be imported from the HDC Service database system. Data on the use of the ChildWeCare system, such as the development levels of the children, the assignments that parents receive, and the results of receiving the service and follow-up care and counseling, will all be updated in the system.

Telehealth technologies, tools, and services are becoming an important component of healthcare systems [34,35,36,37]. The US Department of Health and Human Services estimates that more than 60% of all healthcare institutions and 40–50% of hospitals in the US currently use some form of telehealth technology [34,38]. Late in 2016, Kaiser Permanente of Northern California reported that its virtual (e-mail, telephone, and video) communications exceeded in-person visits [39]. Moreover, the Geisinger Health System, Intermountain Healthcare, Partners HealthCare, the University of Virginia Health System, and the Veterans Health Administration use telehealth interventions for purposes such as filling gaps in care that result from provider shortages and providing access to services after normal clinic hours, thereby reducing patient and family travel burdens, facilitating services such as appointment scheduling and refilling prescriptions, and responding to business challenges and consumer expectations [34,40].

It should be noted that the ChildWeCare program only provides appointment reminders and materials for developmental stimulation and direct answers to queries related to these. Caregivers should still attend appointments with the pediatrician or therapist to assess the developmental needs of their child. Appropriate developmental stimulation can help children develop better skills and abilities in various areas, thereby making them better able to receive a higher education, live on their own, and reduce the burden of care and living expenses of parents. In the long term, the ChildWeCare system will be expanded to cover the whole of Health Area 1 and then be deployed nationwide.

A long wait by a child with developmental problems for a referral to receive appropriate developmental stimulation might make their outcomes worse; thus, appropriate assignments to stimulate child development in the meantime would be advantageous. The outcomes of the pilot testing show that the parents were satisfied with the ChildWeCare system. However, the efficiency of the online stimulation via the program was not examined in this phase. Further large-scale testing is still necessary.

According to the SWOT analysis results for the program, its strengths, such as access being free, its ease of use, and its user-friendly interface, might be advantageous for recruiting the target population. However, some weaknesses and threats should be considered, including a lack of internet access in some settings and some people being unwilling to access the program online. To deal with these problems, we also prepared a paper-based developmental stimulation manual and assistance to help caregivers in these scenarios. In addition, the developmental stimulation scenarios in the program are short and directly communicated, which might address the issue related to some caregivers having insufficient time to travel to and attend appointments at healthcare facilities.

## 5. Limitations and Recommendations

Since this study involved pilot testing of the program prior to its implementation for a larger target group, user satisfaction with and problems arising while using the program were assessed using only a few volunteer participants (the parents of children with developmental delays). Testing with a larger sample and/or in other settings might help uncover any issues that should be handled before a nationwide launch. In addition, the validity and reliability of the program were not determined, both of which are necessary before the future implementation of the program nationwide.

## 6. Conclusions

In this study, we developed and pilot-tested the ChildWeCare system for monitoring and providing care for children with developmental delays from birth to five years old who have already been screened. The pilot test was conducted at an event held in a hospital in northern Thailand. The satisfaction of the parents who used the system in the pilot test was good on average. These preliminary results are encouraging and could lead to more widespread implementation of the system in Health Region 1 (Chiang Mai, Lamphun, Lampang, Phrae, Nan, Phayao, Chiang Rai, and Mae Hong Son) and eventually nationwide.

## Figures and Tables

**Table 1:** The available homework assignments and video clips for parents of children after screening for developmental delays using DSPM.

Set	Age (Months)	Development Domain					Code	Total No. of Assignments	Total No. of Clips	Clip Name
		GM	FM	RL	EL	PS				
1	9	Fail	Fail	Fail	Fail	Fail	a01	5	5	gm9.mp4, fm9.mp4, rl9.mp4, el9.mp4, ps9.mp4
2	9	Fail	Fail	Fail	Fail	Pass	a02	4	4	gm9.mp4, fm9.mp4, rl9.mp4, el9.mp4
3	9	Fail	Fail	Fail	Pass	Fail	a03	4	4	gm9.mp4, fm9.mp4, rl9.mp4, ps9.mp4
4	9	Fail	Fail	Fail	Pass	Pass	a04	3	3	gm9.mp4, fm9.mp4, rl9.mp4
5	9	Fail	Fail	Pass	Fail	Fail	a05	4	4	gm9.mp4, fm9.mp4, el9.mp4, ps9.mp4
. . .										
149	60	Pass	Pass	Fail	Fail	Fail	a25	3	3	rl60.mp4, el60.mp4, ps60.mp4
150	60	Pass	Pass	Fail	Fail	Pass	a26	2	2	rl60.mp4, el60.mp4
151	60	Pass	Pass	Fail	Pass	Fail	a27	2	2	rl60.mp4, ps60.mp4
152	60	Pass	Pass	Fail	Pass	Pass	a28	1	1	rl60.mp4
153	60	Pass	Pass	Pass	Fail	Fail	a29	2	2	el60.mp4, ps60.mp4
154	60	Pass	Pass	Pass	Fail	Pass	a30	1	1	el60.mp4
155	60	Pass	Pass	Pass	Pass	Fail	a31	1	1	ps60.mp4

GM, gross motor skill; FM, fine motor skill; RL, receptive language skill; EL, expressive language skill; PS, personal and social skill.

**Table 2:** List of available homework assignments and video clips for parents of children after screening for developmental delays using TEDA4I.

ID	Age (Months)	Number of Evaluation Questions					Total
		GM	FM	RL	EL	PS	
1	2	1	1	1	1	1	5
2	4	1	1	1	1	1	5
3	6	1	1	1	1	1	5
4	8	1	1	1	1	1	5
5	10	1	1	1	1	1	5
6	12	2	2	2	2	2	10
7	15	1	2	2	2	2	9
8	18	1	1	2	2	2	8
9	21	1	1	1	2	2	7
10	24	1	2	2	2	2	9
11	30	1	2	2	2	2	9
12	36	2	2	2	2	2	10
13	42	2	2	2	2	2	10
14	48	2	2	2	2	2	10
15	54	2	2	2	2	2	10
16	60	2	2	2	2	2	10
17	66	2	2	1	2	2	9
18	72	1	2	2	2	2	9
Total		25	29	29	31	31	145

GM, gross motor skill; FM, fine motor skill; RL, receptive language skill; EL, ex-pressive language skill; PS, personal and social skill.

**Table 3:** Examples of homework assignments for parents after their child’s assessment with the TEDA4I tool.

Parent ID	Real Age (Months)	Developmental Age (Months)					Homework Assignments				
		GM	FM	RL	EL	PS	GM	FM	RL	EL	PS
1	12	10	8	6	6	6	6, 7	5	4	4	4
2	20	15	12	12	12	10	9	8, 9	8, 9	8, 9	6, 7
3	36	24	24	24	24	21	12	14, 15	15, 16	16, 17	14, 15

**Table 4:** Details of the children who participated in the pilot testing of the ChildWeCare system at Thoen Hospital (N = 7).

ID	Gender	Real Age (Months)	Developmental Age (Months) ^a^				
			LGM	LFM	LRL	LEL	LPS
1	Female	60	42	21	10	12	30
2	Male	26	30	36	24	30	42
3	Male	27	30	21	21	15	18
4	Female	41	15	21	30	30	24
5	Male	42	18	21	12	18	18
6	Male	63	18	36	12	21	36
7	Female	93	21	30	30	36	48

LGM, late gross motor; LFM, late fine motor; LRL, late receptive language (LRL); LEL, late expressive language; LPS, late personal and social. ^a^ The developmental age of the child as assessed by healthcare professionals.

**Table 5:** Assignment workflow in the pilot testing of the ChildWeCare system at Thoen Hospital (N = 7).

ID	Child’s Age (Months)	Relationship of the Caregiver	Educational Level	Registered Status	Assignment Workflow
1	60	Grandmother	Primary school	Completed	Completed
2	26	Mother	Secondary school	Completed	Completed
3	27	Mother	Diploma	Completed	Completed
4	41	Caregiver	Primary school	Completed	Completed
5	42	Grandmother	Primary school	Completed	Completed
6	63	Grandmother	High school	Completed	Completed
7	93	Mother	Bachelor’s degree	Completed	Completed

**Table 6:** SWOT analysis of the ChildWeCare system.

Strengths	Weaknesses	Opportunities	Treats
Easy to connect and register via the online application	The developmental age is not updated in real-time	The number of internet users is continuously increasing, making it easier to reach more children with developmental delays lost to follow-up	Internet access might not cover all areas of Thailand, especially in the northern region located in a mountainous setting
Short video developmental stimulation assignments can be directly accessed by the caregivers	Answers to queries are only available on business days	The use of telehealth services has increased in Thailand since the COVID-19 outbreak, so the participants were more likely to be familiar with them	Some caregivers, such as older adults, may not be comfortable using the program
The program has two-way communication: the caregivers can consult with the staff and the staff can directly contact the caregivers			The caregivers may be too busy to answer questions and complete assignments
The program is free to access			
Reduces problems associated with long travel distances and/or costs			

## Data Availability

Data will be made available upon appropriate request to the corresponding author due to privacy and ethical restrictions.
